# The Story of the Insane from Year to Year

**Published:** 1905-12-30

**Authors:** 


### THE STORY OF THE INSANE FROM YEAR TO YEAR.

SEVENTEENTH ANNUAL SERIES.

### THE JAMAICA ASYLUM.

AT the beginning of the year this Asylum contained 959 patients, and at the end of the year the number had risen to 1,000. There were 146 first admissions, 60 readmissions, 67 recoveries, 3 discharged not improved, and 95 died. The average number resident during the year was 972, and the total number under treatment was 1,165. We notice that the year's admissions were made up of 4 white men, 52 brown, 144 black, 4 were coolies, and 2 were Chinese. For the sake of comparison with other asylums, we could wish that a few, at any rate, of the tables of the Medico-Psychological Association were published in this report. To be told that heredity and stress are the chief causes of insanity in the island is hardly enough, as we should like to be informed as to the types of insanity most prevalent among the somewhat mixed population over which Dr. Williams presides. The recoveries stand at 32.52 per cent. of the year's admissions, and the deaths at 8.15 per cent. of the total number under treatment. We regret to hear of the death of Dr. Plaxton, who for eighteen years had held the post of Medical Superintendent of the Asylum. "During his long tenure of office he devoted himself to the discharge of his arduous duties with unremitting attention, and by his death the Institution has suffered a severe loss." Dr. Dowden resigned the post of junior assistant medical officer, and Dr. E. M. Thompson was appointed to fill the vacancy. The weekly cost per head was 6s. 2d.

